# A secretion biosensor for monitoring Sec-dependent protein export in *Corynebacterium glutamicum*

**DOI:** 10.1186/s12934-019-1273-z

**Published:** 2020-01-21

**Authors:** Sarah Jurischka, Astrid Bida, Doris Dohmen-Olma, Britta Kleine, Janko Potzkei, Stephan Binder, Georg Schaumann, Patrick J. Bakkes, Roland Freudl

**Affiliations:** 1 Institut für Bio- und Geowissenschaften 1, IBG1: Biotechnologie, Forschungszentrum Jülich GmbH, 52425 Jülich, Germany; 2 Bioeconomy Science Center (BioSC), 52425 Jülich, Germany; 3 SenseUp GmbH, c/o Campus Forschungszentrum, Wilhelm-Johnen-Strasse, 52428 Jülich, Germany

**Keywords:** *Corynebacterium glutamicum*, Heterologous protein production, Sec pathway, Secretion biosensor, Signal peptide variation, FACS analysis

## Abstract

**Background:**

In recent years, the industrial workhorse *Corynebacterium glutamicum* has gained increasing interest as a host organism for the secretory production of heterologous proteins. Generally, the yield of a target protein in the culture supernatant depends on a multitude of interdependent biological and bioprocess parameters which have to be optimized. So far, the monitoring of such optimization processes depends on the availability of a direct assay for the respective target protein that can be handled also in high throughput approaches. Since simple assays, such as standard enzymatic activity assays, are not always at hand, the availability of a general protein secretion biosensor is highly desirable.

**Results:**

High level secretion of proteins via the Sec protein export pathway leads to secretion stress, a phenomenon that is thought to be caused by the accumulation of incompletely or misfolded proteins at the membrane-cell envelope interface. We have analyzed the transcriptional responses of *C. glutamicum* to the secretory production of two different heterologous proteins and found that, in both cases, the expression of the gene encoding a homologue of the extracytosolic HtrA protease was highly upregulated. Based on this finding, a *C. glutamicum* Sec secretion biosensor strain was constructed in which the *htrA* gene on the chromosome was replaced by the *eyfp* gene. The fluorescence of the resulting reporter strain responded to the secretion of different heterologous proteins (cutinase from *Fusarium solani pisi* and alkaline phosphatase PhoA from *Escherichia coli*) in a dose-dependent manner. In addition, three differently efficient signal peptides for the secretory production of the cutinase could be differentiated by the biosensor signal. Furthermore, we have shown that an efficient signal peptide can be separated from a poor signal peptide by using the biosensor signal of the respective cells in fluorescence activated cell sorting experiments.

**Conclusions:**

We have succeeded in the construction of a *C. glutamicum* biosensor strain that allows for the monitoring of Sec-dependent secretion of heterologous proteins in a dose-dependent manner, independent of a direct assay for the desired target protein.

## Background

Biotechnologically or pharmaceutically relevant recombinant proteins represent a steadily increasing multi-billion dollar market, and for their production, various different pro- and eukaryotic expression hosts are currently used. Herein, in many cases, the secretion of the respective target proteins into the culture supernatant of an expression host represents an attractive alternative strategy to intracellular production, since product recovery is greatly simplified and, as a consequence, the production costs can be significantly reduced [1].

The diderm Gram-positive bacterium *Corynebacterium glutamicum* is an industrial workhorse that has a long tradition in industry as a producer organism for various amino acids and other low-molecular weight compounds [2, 3]. In recent years however, it became clear that *C. glutamicum* also has a huge potential as a host organism for the secretory production of heterologous proteins [4]. In contrast to many other commonly used bacterial secretory production hosts, such as various *Bacillus* species, *C. glutamicum* secretes only a very limited number of endogenous proteins into its culture supernatant and exhibits very low, if any extracellular proteolytic activity, making this microorganism very attractive for the secretion of even protease-sensitive heterologous proteins. Therefore, the secreted proteins of interest are proteolytically stable and are present in a very high relative purity in the respective fermentation media [5].

Like in other bacteria, also in *C. glutamicum* the great majority of extracytosolic proteins are transported out of the cytosol via the general secretion (Sec) protein export system [4, 6]. Sec substrates are synthesized as precursor proteins that possess an amino-terminal signal peptide that is responsible for the targeting of the proteins to the Sec translocase located in the cytoplasmic membrane [7]. Subsequently, the proteins destined for export are translocated across the membrane in an unfolded state through a pore formed by the SecYEG complex [8]. During or shortly after membrane translocation, the signal peptide is removed by signal peptidase [9] and the mature part of the protein is released on the *trans*-side of the membrane where the folding of the protein into its native conformation takes place.

The final yield of a heterologous target protein in the culture supernatant of the bacterial secretory production host strongly depends on a multitude of interdependent biological and bioprocess parameters [10]. For instance, the identity of the signal peptide that is used to drive the Sec-dependent membrane translocation of the target protein has been shown to be one of the critical parameters that are decisive whether a production process becomes successful and economically relevant or not [11, 12]. Furthermore, process conditions such as medium composition, inducer concentration, induction time, temperature, and substrate feed rates also strongly influence the amounts of a desired target protein in the culture supernatant of the respective expression host [10, 13, 14]. Since the number of possible parameter combinations exponentially grows with every additional parameter, the testing of a huge number of conditions is desirable to achieve the optimal yields for each individual target protein. At present, the monitoring of such an optimization process relies heavily on the availability of a direct assay for the respective target protein. However, simple assays, such as standard enzymatic activity assays, are not always at hand. For example, the quantification of pharmaceutical proteins often requires elaborate biological activity assays, which limits the number of samples that can be tested during the process optimization procedure. Therefore, process optimization would greatly benefit from the availability of a general secretion biosensor that can be handled in high throughput approaches and that allows for an assay-independent monitoring of target protein secretion in an efficient and dose-dependent manner.

The overproduction of exported proteins leads to secretion stress, a phenomenon that is thought to be caused by the accumulation of incompletely or misfolded proteins at the membrane-cell envelope interface [15]. A highly conserved cellular response to this secretion stress is the upregulation of extracytosolic proteases, such as HtrA/DegP, that counteract this stress by degrading the unfolded proteins at the *trans*-side of the membrane [16]. For the Gram-positive model organism *Bacillus subtilis*, it has been shown that the two component system CssRS senses the secretion stress and activates the expression of the *htrA* and *htrB* genes, resulting in increased amounts of the quality control proteases HtrA and HtrB, respectively [17, 18]. Based on the respective sensing system, a reporter strain was constructed in which the *gfp* gene was placed under the control of the promoter of the *htrA* gene and the respective strain was used for the identification of secreted proteins by fluorescence activated cell sorting (FACS) after expressing a partial genomic library from the plant root-colonizing bacterium *Bacillus amyloliquefaciens* [19].

In the present manuscript, we have analyzed the transcriptional responses of *C. glutamicum* to the secretory production of two different heterologous proteins, i.e. an α-amylase (AmyE) from *B. subtilis*, and a cutinase from the fungus *Fusarium solani pisi*, by microarray experiments. Two major stress responses, i.e. (1) the induction of the EsrISR cell envelope stress response system [20] and (2) the upregulation of the gene encoding a homologue of the HtrA protease were observed. For the construction of a *C. glutamicum* Sec secretion biosensor, we replaced the *htrA* gene on the chromosome by the *eyfp* gene and, in fact, the eYFP fluorescence of the resulting *C. glutamicum* reporter strain responded to the secretion of different heterologous proteins in a dose-dependent manner. Next, we fused three different Sec signal peptides to the heterologous model protein cutinase from *F. solani pisi* and produced the corresponding hybrid precursor proteins in *C. glutamicum*. The amounts of cutinase in the respective culture supernatants varied significantly, depending on the signal peptide that was used to drive Sec-dependent membrane transport of the cutinase. Importantly, the fluorescence of the Sec secretion biosensor in the respective cutinase secreting cells reflected the amount of cutinase that was secreted into the culture supernatant of the corresponding cells. Using mixtures of two strains that secreted cutinase with different efficiencies, we subsequently showed that the cells producing the cutinase with the superior signal peptide can be sorted out by FACS, demonstrating the suitability of the Sec secretion biosensor for the high throughput optimization of secretory protein production by *C. glutamicum*.

## Results

### Identification of Sec secretion stress-responsive genes in *C. glutamicum*

For the identification of Sec secretion stress-responsive genes in *C. glutamicum*, two heterologous secretory model precursor proteins, *i.e.* a cutinase from the fungus *F. solani pisi* fused to the *B. subtilis* NprE signal peptide [10] and the α-amylase AmyE from *B. subtilis* containing its authentic signal peptide [21] and a carboxyl-terminal His_6_-tag were used. The corresponding genes were cloned into the expression vector pEKEx2 under the regulatory control of the *tac* promoter, allowing an IPTG-inducible expression in *C. glutamicum*. After transformation of *C. glutamicum* with the respective plasmids, the transcriptome of cells that were fully induced by the addition of 1 mM IPTG was compared with the transcriptome of uninduced cells that only show a basal expression of the corresponding genes and a low level secretion of the two heterologous model proteins (Additional file 1: Figure S1). To differentiate the cellular responses that are caused by the expression of a heterologous protein per se (such as the upregulation of the genes encoding the general chaperone systems GroELS and DnaK/DnaJ/GrpE) from those that are caused by the secretion of the protein across the cytoplasmic membrane, we also included *C. glutamicum* cells that expressed a signal peptide-less and therefore cytosolic variant (Additional file 1: Figure S1) of the His_6_-tagged AmyE protein (ΔSP-AmyE) in our microarray analyses.

In Table 1, a selection of several genes is shown that were specifically upregulated more than twofold in the *C. glutamicum* strain overproducing and secreting AmyE, but not in the strain producing the signal peptide-less AmyE variant. Three of these genes, *esrI* (cg0706), cg1325, and *rsmP* (cg3264), belong to envelope stress response (Esr) regulon that is regulated by the EsrISR three component system. The Esr regulon is induced under conditions that threaten the integrity of the cell envelope and it can be activated, among others, by antibiotics that inhibit the lipid II cycle, such as bacitracin and vancomycin [20]. Another strong secretion-specific upregulation upon AmyE overproduction (i.e. 4.22 fold) was observed for the *htrA* (cg0998) gene. A similar upregulation (4.40 fold) of the *htrA* gene, but not of the genes belonging to the Esr regulon, was observed when the *F. solani pisi* cutinase was secreted via the Sec pathway using the NprE signal peptide (Table 1). Taken together, these results indicate that, like in other microorganisms [22–24], the upregulation of the extracytosolic protease HtrA is a common stress response that also in *C. glutamicum* counteracts secretion stress by degrading accumulated misfolded proteins at the outer surface of the cytoplasmic membrane.

**Table 1:** *C. glutamicum* genes responding to Sec secretion stress in microarray experiments

Gene locus	Gene	Annotation	Average mRNA ratio ΔSP-AmyE overproduction^a^	Average mRNA ratio AmyE overproduction^b^	Average mRNA ratio NrpE-cutinase overproduction^c^
cg0998	*htrA*	HtrA protease homologue	1.24	*4.22*	*4.40*
cg0706	*esrI*	EsrI, inhibitor of the EsrSR two-component system	0.99*	*2.31*	0.80*
cg1325		Putative stress responsive transcriptional regulator	1.13*	*3.69*	0.96*
cg3264	*rsmP*	RsmP, conserved cytoskeletal protein	0.61	*2.67*	0.96*

Significant (p ≤ 0.05) more than twofold changes in the average mRNA ratios in the DNA microarray experiments are indicated in italic numbers. Average mRNA ratios with p > 0.05 are marked by an asterisk. All microarray data were from three independent biological samples

^a^Average mRNA ratio of *C. glutamicum* (pEKEx2-ΔSP-AmyE) induced/uninduced by IPTG for 30 min

^b^Average mRNA ratio of *C. glutamicum* (pEKEx2-AmyE) induced/uninduced by IPTG for 30 min

^c^Average mRNA ratio of *C. glutamicum* (pEKEx2-NprE-cutinase) induced/uninduced by IPTG for 30 min

### Construction of a Sec secretion stress-responsive *C. glutamicum* biosensor strain

**Fig. 1:**
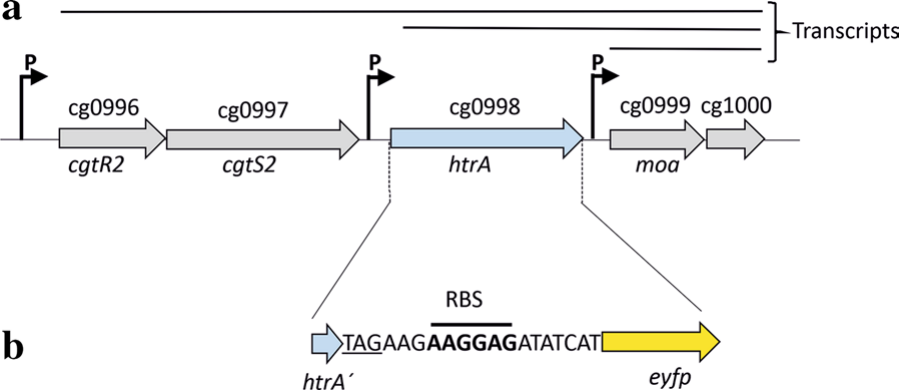
The *htrA* locus of *C. glutamicum*. **a** Genomic organization of the chromosomal *cgtR2*-*cgtS2*-*htrA*-*moa*-cg1000 locus in *C. glutamicum* ATCC13032 wild-type. The promoters (P) upstream and within the operon and the transcripts derived from the respective promoters [25] are indicated. **b** In *C. glutamicum* K9, the *htrA* gene was replaced by a DNA fragment encompassing the first 51 bp of *htrA* (*htrA*′) followed by a TAG stop codon (underlined), a 16 bp spacer containing a ribosome binding site (RBS, bold letters in the nucleotide sequence) and the *eyfp* gene

Since *htrA* gene expression was increased upon secretory production of two different heterologous proteins, we reasoned that placing the gene encoding eYFP under the respective regulatory control should result in a fluorescent biosensor that is able to monitor the extent of protein secretion in *C. glutamicum*. As shown in Fig. 1a, *htrA* is the third gene in an operon consisting of the genes *cgtR2*–*cgtS2*–*htrA*–*moa*–cg1000. *cgtR2* and *cgtS2* code for the response regulator and the sensor kinase of a two-component system which, similar to the situation in *Mycobacterium tuberculosis*, might be involved in the stress-responsive regulation of the adjacent *htrA* gene [23]. Downstream of *htrA*, a gene (*moa*) encoding a predicted molybdopterin biosynthetic protein and a small gene (cg1000) for a hypothetical gene product of unknown function are located in the operon. According to the RNAseq analysis of *C. glutamicum* transcriptomes [25], a polycistronic mRNA encompassing all five genes of the operon is transcribed from one or possibly two promoters upstream of the *cgtR2* gene. In addition, an mRNA encompassing *htrA*-*moa*-cg1000 is transcribed from a promoter located directly upstream the *htrA* gene. A third transcript starts from a promoter located in front of *moa* and encompasses the last two genes (*moa*-cg1000) from the operon. The detailed mechanism of the stress-responsive upregulation of *htrA* expression in *C. glutamicum* is unknown so far.

**Fig. 2:**
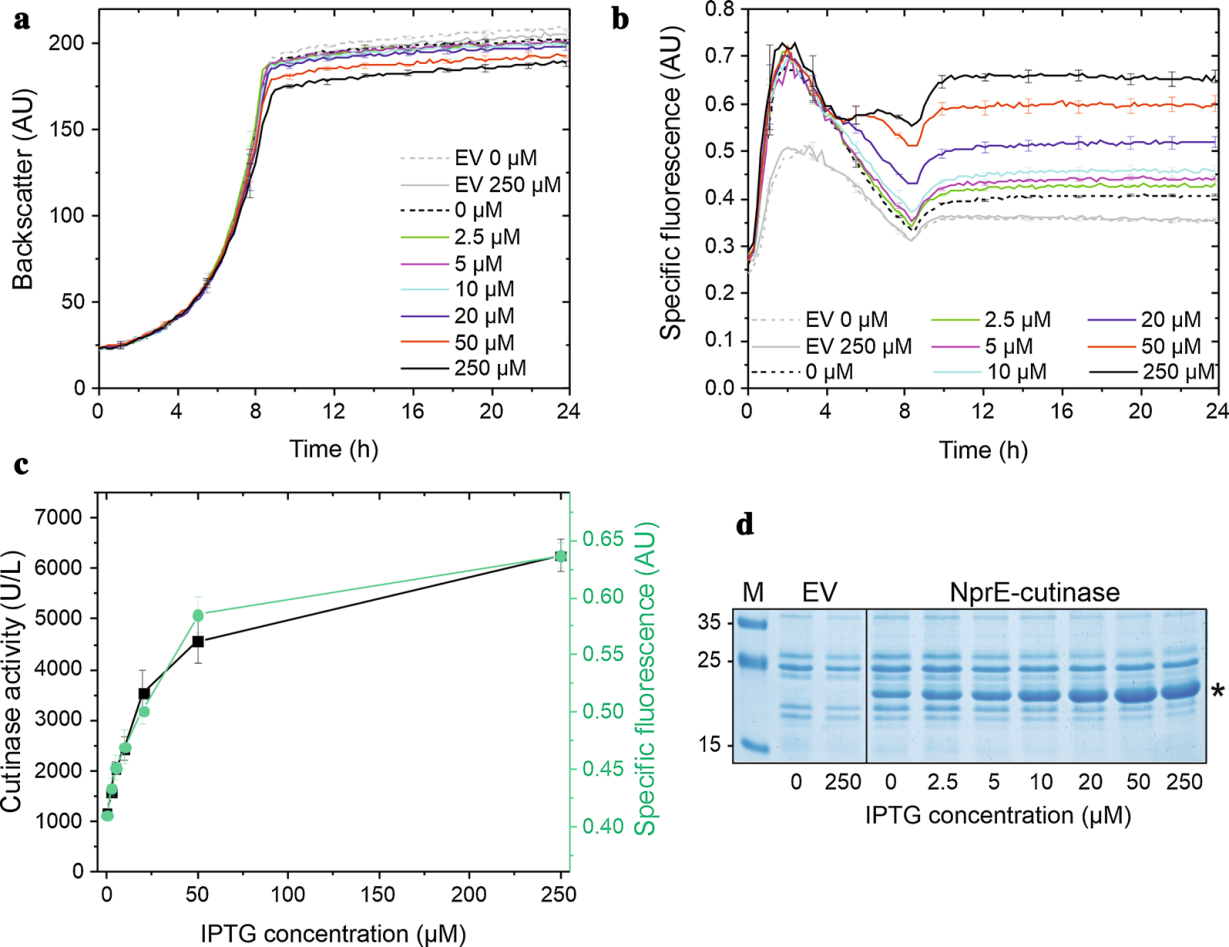
Cutinase secretion by *C. glutamicum* K9. Cells of *C. glutamicum* K9 possessing the pEKEx2 empty vector (EV) or pEKEx2-NprE-cutinase were inoculated to an OD_600_ of 0.5 in 750 µl CGXII medium in a 48-well FlowerPlate and subsequently cultivated in a BioLector for 24 h at 30 °C, 1200 rpm and constant 85% relative humidity. After 4 h of cultivation, IPTG was added at the indicated final concentrations. **a** Growth of the respective cultures was monitored as backscattered light in 15 min intervals starting at the beginning of the cultivation. The growth curves show one representative experiment of three independent biological replicates. Standard deviations are given for selected time points. **b** Specific fluorescence of the respective cultures during the BioLector cultivation. Also here, one representative experiment of three independent biological replicates is shown and the standard deviations are given for selected time points. **c** Cutinase activity in the supernatant (black symbols) and specific fluorescence values (green symbols) after 24 h of cultivation of *C. glutamicum* K9 (pEKEx2-NprE-cutinase) induced by different IPTG concentrations. **d** After 24 h of growth, samples of the culture supernatant corresponding to an equal number of the respective *C. glutamicum* K9 cells that had been induced by the IPTG concentrations indicated below the lanes were analyzed by SDS-PAGE and proteins were visualized by Coomassie Brilliant Blue staining. EV, *C. glutamicum* K9 (pEKEx2 empty vector) as negative control; NprE-cutinase, *C. glutamicum* K9 (pEKEx2-NprE-cutinase); M, molecular weight protein markers in kDa. The position of the secreted cutinase protein is indicated by an asterisk. *AU* arbitrary units

For the construction of a Sec secretion stress-responsive biosensor strain, we replaced the *htrA* gene on the chromosome of *C. glutamicum* ATCC13032 by a DNA fragment encompassing the first 51 nucleotides of the *htrA* structural gene followed by a TAG stop codon, a 16 bp spacer sequence containing a ribosome binding site, and the *eyfp* gene (Fig. 1b). The resulting *C. glutamicum* strain K9 was subsequently transformed with pEKEx2-NprE-cutinase and cultivated in a BioLector device that allows the online monitoring of cell growth and fluorescence [26] in the absence or presence of different concentrations of IPTG for the induction of NprE-cutinase expression. As a control, *C. glutamicum* K9 containing the pEKEx2 empty vector was also included in the analysis. As shown in Fig. 2a, the growth of the various strains was similar, although a slight growth defect, the extent of which depended on the IPTG concentrations used, could be observed in *C. glutamicum* K9 (pEKEx2-NprE-cutinase) compared with *C. glutamicum* K9 containing the pEKEx2 empty vector. This is fully in line with the known fact that recombinant protein production and secretion causes a metabolic burden to bacterial cells which results in growth reduction [27, 28]. Remarkably, the control strain K9 containing the pEKEx2 empty vector already shows a significant specific fluorescence (i.e. 0.35 AU at 24 h of cultivation) with no apparent effect of IPTG addition (Fig. 2b). In contrast, *C. glutamicum* per se does not possess any significant intrinsic fluorescence (Additional file 1: Figure S2). Since the *eyfp* gene in the control strain is under the control of the native regulatory elements of the *htrA* gene whose expression responds to the existing secretion stress, the fluorescence of the control strain likely reflects the basal secretion stress level that is exerted by the Sec-dependent export of host-derived proteins across the cytoplasmic membrane. Importantly, the additional expression and secretion of NprE-cutinase resulted in a significant increase of the specific fluorescence values above the basal level, whereby increasing amounts of IPTG resulted in correspondingly increasing specific fluorescence values (Fig. 2b). Since with increasing IPTG concentrations, increasing cutinase activities (Fig. 2c) and increasing amounts of cutinase protein (Fig. 2d) are observed in the respective culture supernatants, our results indicate that the K9 secretion biosensor fluorescence signal reflects the amount of cutinase that is secreted across the cytoplasmic membrane into the culture supernatant. Indeed, there is a very good correlation between the cutinase activity in the supernatant determined at the end of the BioLector cultivation (at 24 h) and the specific fluorescence measured at the same time point (Fig. 2c).

**Fig. 3:**
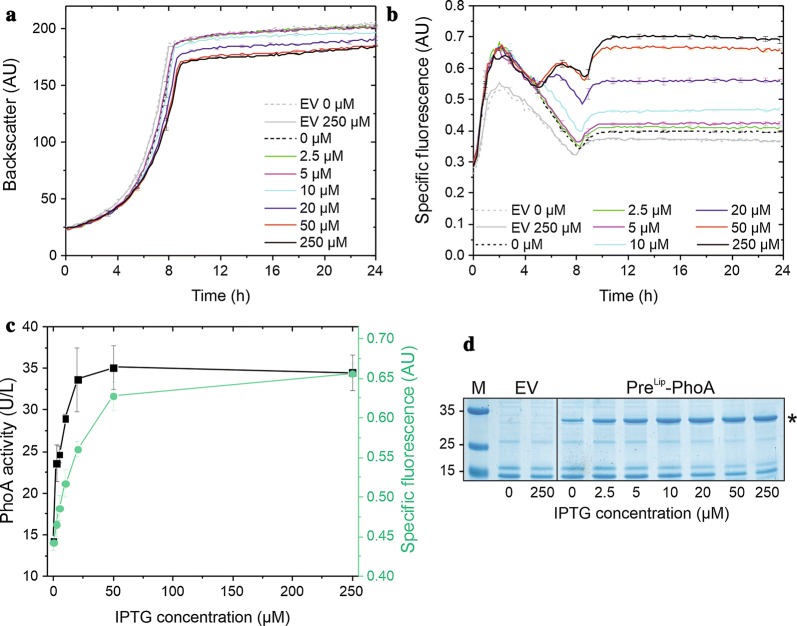
PhoA secretion by *C. glutamicum* K9. Cells of *C. glutamicum* K9 possessing the pEKEx2 empty vector (EV) or pEKEx2-Pre^Lip^-PhoA were inoculated to an OD_600_ of 0.5 in 750 µl CGXII medium in a 48-well FlowerPlate and subsequently cultivated in a BioLector for 24 h at 30 °C, 1200 rpm and constant 85% relative humidity. After 4 h of cultivation, IPTG was added at the indicated final concentrations. **a** Growth of the respective cultures was monitored as backscattered light in 15 min intervals starting at the beginning of the cultivation. The growth curves show one representative experiment of three independent biological replicates. Standard deviations are given for selected time points. **b** Specific fluorescence of the respective cultures during the BioLector cultivation. Also here, one representative experiment of three independent biological replicates is shown and the standard deviations are given for selected time points. **c** PhoA activity in the supernatant (black symbols) and specific fluorescence values (green symbols) after 24 h of cultivation of *C. glutamicum* K9 (pEKEx2-Pre^Lip^-PhoA) induced by different IPTG concentrations. **d** After 24 h of growth, samples of the culture supernatant corresponding to an equal number of the respective *C. glutamicum* K9 cells that had been induced by the IPTG concentrations indicated below the lanes were analyzed by SDS-PAGE and proteins were visualized by Coomassie Brilliant Blue staining. EV, *C. glutamicum* K9 (pEKEx2 empty vector) as negative control; Pre^Lip^-PhoA, *C. glutamicum* K9 (pEKEx2-Pre^Lip^-PhoA); M, molecular weight protein markers in kDa. The position of the secreted PhoA protein is indicated by an asterisk. *AU* arbitrary units

We next analyzed the behaviour of the K9 Sec secretion biosensor in response to the secretion of another, completely unrelated heterologous model protein, i.e. the alkaline phosphatase PhoA of *E. coli*. For the channeling of PhoA into the *C. glutamicum* Sec protein export pathway, we used the signal peptide (Pre^Lip^) of the *Staphylococcus hyicus* lipase [29]. Previous experiments with *B. subtilis* [30] and also with *C. glutamicum* (our observations) have indicated that PhoA is secreted only very poorly with its native Sec signal peptide in the respective foreign Gram-positive hosts and that efficient secretion of PhoA can be achieved when the mature PhoA protein is fused to the lipase-derived signal peptide. *C. glutamicum* K9 was transformed with plasmid pEKEx2-Pre^Lip^-PhoA and the respective strain was cultivated in the BioLector device in the absence or presence of different IPTG concentrations. Similar to the situation with NprE-cutinase, increasing amounts of IPTG resulted in a concomitant slight decrease in growth (Fig. 3a), reflecting the metabolic burden due to increasing recombinant protein production and secretion. Furthermore, increasing concentrations of IPTG resulted in a corresponding increase in the specific fluorescence of the respective cultures (Fig. 3b), in increasing alkaline phosphatase activities (Fig. 3c), and in increasing amounts of secreted PhoA protein in the culture supernatant (Fig. 3d). Based on these findings, we conclude that we have succeeded in the construction of a functional Sec secretion biosensor that is able to monitor the extent of secretion of different heterologous proteins in *C. glutamicum*.

### Differently efficient signal peptides for cutinase secretion in *C. glutamicum* can be distinguished by the K9 secretion biosensor signal

**Fig. 4:**
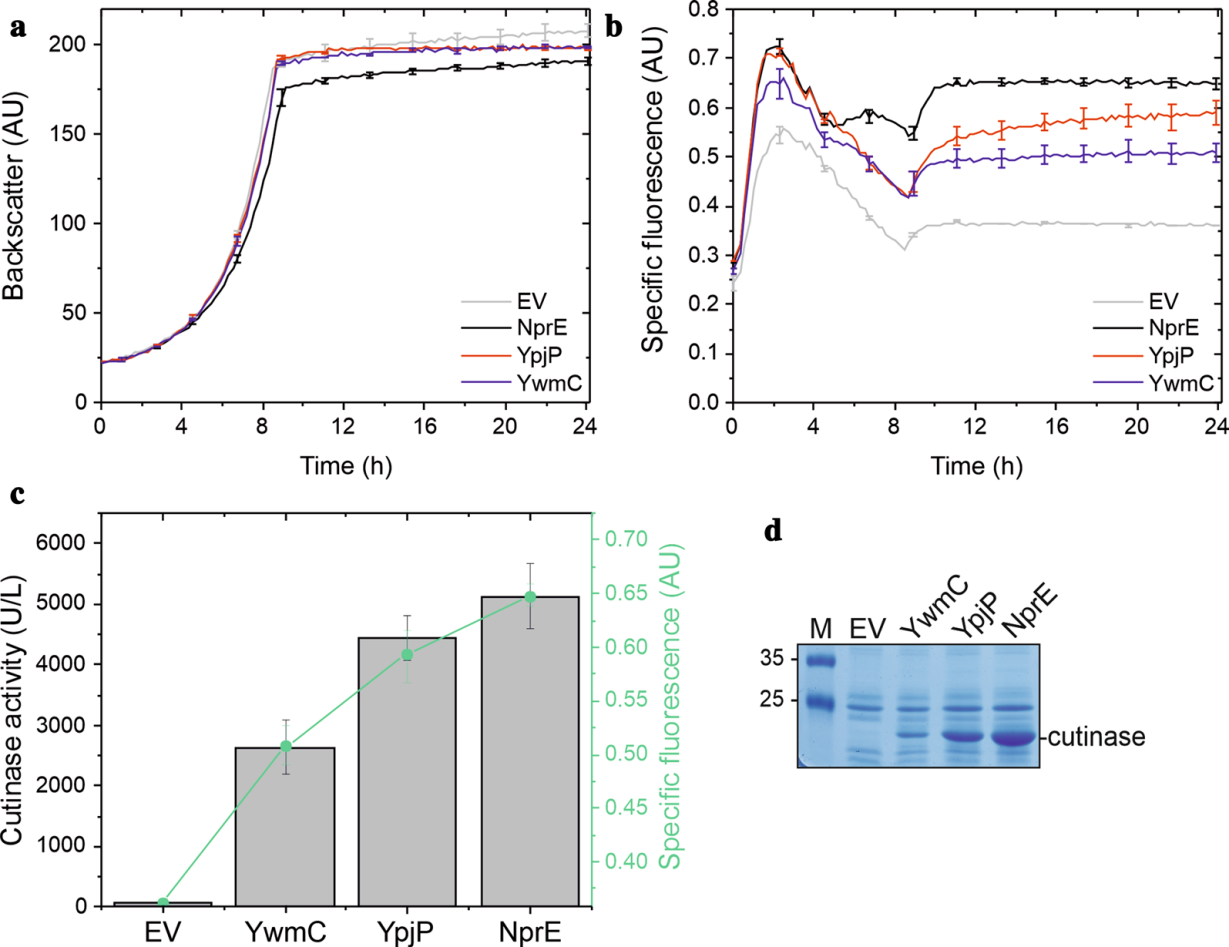
Cutinase secretion by *C. glutamicum* K9 using three different Sec signal peptides. Cells of *C. glutamicum* K9 possessing the pEKEx2 empty vector (EV), pEKEx2-NprE-cutinase (NprE), pEKEx2-YpjP-cutinase (YpjP), or pEKEx2-YwmC-cutinase (YwmC) were inoculated to an OD_600_ of 0.5 in 750 µl CGXII medium in 48-well FlowerPlates and subsequently cultivated in a BioLector system for 24 h at 30 °C, 1200 rpm and constant 85% relative humidity. After 4 h of cultivation, IPTG was added (250 µM final concentration). **a** Growth of the respective cultures was monitored as backscattered light in 15 min intervals starting at the beginning of the cultivation. The growth curves show one representative experiment of three independent biological replicates. Standard deviations are given for selected time points. **b** Specific fluorescence of the respective cultures during the BioLector cultivation. Also here, one representative experiment of three independent biological replicates is shown and the standard deviations are given for selected time points. **c** Cutinase activity in the supernatant (gray bars) and specific fluorescence values (green dots) after 24 h of cultivation. **d** After 24 h of growth, samples of the culture supernatant corresponding to an equal number of the respective *C. glutamicum* K9 cells were analyzed by SDS-PAGE and the proteins were visualized by Coomassie Brilliant Blue staining. M, molecular weight protein markers in kDa. The position of the secreted cutinase protein is indicated. *AU* arbitrary units

In recent years, it has become increasingly clear that the choice of the signal peptide that is used to direct a desired heterologous protein into the Sec export pathway is one of the most critical steps on the way to an efficient secretory production process. Since so far it is not possible to predict which signal peptide will perform best in the context of a given heterologous target protein, the most promising way to find the optimal signal peptide is to screen a large diversity of signal peptides, either generated by signal peptide variation using signal peptide libraries or, alternatively, by optimization of a selected signal peptide by random mutagenesis approaches [12]. Since the K9 secretion biosensor responded to cutinase and PhoA secretion in a dose-dependent manner, we next investigated if the K9 secretion biosensor can be used to distinguish between differently efficient signal peptides with respect to secretory protein production by *C. glutamicum*. For this purpose, a set of three cutinase precursor proteins possessing three selected signal peptides from *B. subtilis*, i.e. those derived from the secreted proteins NprE, YpjP, and YwmC [10], was chosen and the corresponding plasmids encoding the different precursor proteins were electroporated into the *C. glutamicum* K9 secretion biosensor strain. The respective strains were cultivated in the BioLector device in the presence of 250 µM IPTG and analyzed with respect to growth, biosensor fluorescence, cutinase protein production, and cutinase activity in the culture supernatant. As shown in Fig. 4, the three signal peptides mediated the secretion of different amounts of cutinase protein into the culture supernatant of the respective *C. glutamicum* K9 strains (Fig. 4d), with corresponding different cutinase activities (Fig. 4c). Herein, the relative efficiency of the signal peptides with respect to cutinase secretion followed the order of NprE > YpjP > YwmC. The relative secretion efficiency mediated by the different signal peptides was also reflected in the growth behaviour of the corresponding recombinant strains, with *C. glutamicum* K9 expressing the NprE-cutinase showing the strongest and *C. glutamicum* K9 expressing the YwmC-cutinase showing the weakest growth defect compared to the *C. glutamicum* K9 control strain harboring the pEKEx2 empty vector (Fig. 4a). Importantly, the relative secretion performance governed by the different signal peptides was also reflected by the specific fluorescence of the corresponding *C. glutamicum* K9 secretion biosensor strains (Fig. 4b, c). From these results, we conclude that signal peptides that facilitate secretion with different efficacies can be distinguished by their respective K9 secretion biosensor signals.

### Biosensor-based signal peptide screening using fluorescence-activated cell sorting (FACS)

**Fig. 5:**
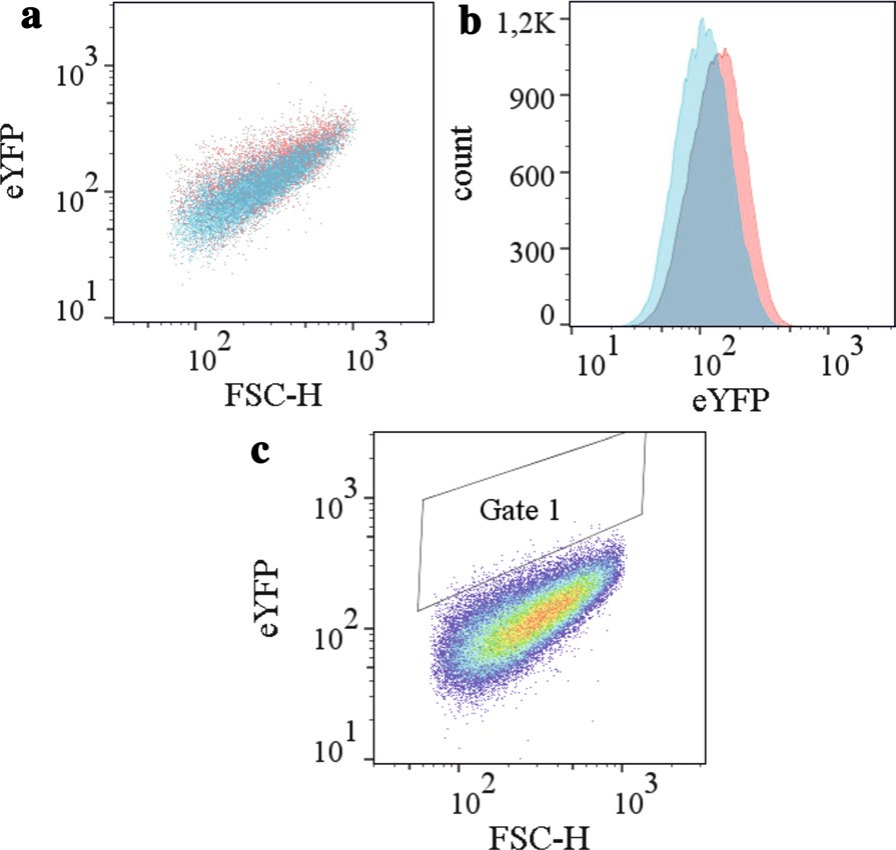
FACS analysis of *C. glutamicum* K9 strains and sorting strategy for the enrichment of the best performing signal peptide. 10^5^ cells from cultures of the respective strains were analyzed, followed by a preselection of cells to exclude cell doublets and debris by electronic gating using FSC-W against FSC-H (Table 2, gate 0). An overlay of *C. glutamicum* K9 carrying pEKEx2-NprE-cutinase (red) or pEKEx2-YwmC-cutinase (blue) is shown as dot plot (**a**) and histogram (**b**). In the dot plot, the fluorescence intensity (eYFP) is plotted against cell size (FSC-H), whereas the histogram shows the cell count against the fluorescence intensity (eYFP). **c** Cells were inoculated to an OD_600_ of 0.5 in CGXII medium containing 2% (w/v) glucose and cultivated at 30 °C. After 4 h of growth, IPTG (250 µM final concentration) was added to the cultures and, after 10 h of growth, cells were sampled from the respective cultures and subjected to FACS analysis. For the enrichment of the better performing signal peptide (NprE) out of a 1:1 or a 1:100 mixture with a less efficient signal peptide (YwmC), we selected a gate (gate 1) in the dot plot of the respective preselected cells such that it contains as many of the better performing cells (i.e. those containing pEKEx2-NprE-cutinase) and excludes as many of the less productive cells (i.e. those containing pEKEx2-YwmC). For the corresponding numbers of events falling in gate 1, see Table 2 and Additional file 1: Figure S4

Next, we investigated whether the secretion biosensor can be exploited in conjunction with fluorescence-activated cell sorting (FACS) to separate and sort cells with different secretion performances, i.e. to discriminate between a potent signal peptide (e.g. NprE) and a poor signal peptide (e.g. YwmC). A graphical workflow of the general setup for the FACS analysis and sorting experiments is shown in Additional file 1: Figure S3. First, the *C. glutamicum* K9 secretion biosensor strains containing either pEKEx2-NprE-cutinase or pEKEx2-YwmC-cutinase were cultivated separately and analyzed by FACS. In a typical experiment, first 10^5^ cells from cultures of the respective strains were analyzed, followed by a preselection of cells to exclude cell doublets and debris by electronic gating using FSC-W against FSC-H (Table 2, gate 0). The respective strains exhibited rather small differences in fluorescence output, as illustrated by the overlay plots shown in Fig. 5a, b. Cells secreting NprE-cutinase exhibited a median fluorescence of 124, which is 12.1% higher than that of cells secreting YwmC-cutinase. These results indicate that the secretion biosensor strain, in principle, can be used to distinguish between between NprE-cutinase- and YwmC-cutinase-secreting cells.

**Table 2:** FACS analysis of cutinase-secreting *C. glutamicum* K9 strains

Signal peptide	Events in gate 0	Events in gate 1	Ratio NprE: YwmC	Percentage NprE (%)
A:				
Before enrichment				
NprE	68,173	309		
YwmC	69,699	7		
Mixture NprE:YwmC	68,915	87	1:1	50
After enrichment				
Enriched culture	68,199	148		
44 clones sequenced			1:0.023 (43 NprE:1 YwmC)	97.7
B:				
Before enrichment				
NprE	71,804	458		
YwmC	71,923	46		
Mixture NprE: YwmC	72,151	50	1:100	1
After enrichment				
Enriched culture	76,932	286		
43 clones sequenced			1:1.7 (16 NprE:27 YwmC)	37

The strains used in the experiments were *C. glutamicum* K9 (pEKEx2-NprE-cutinase), *C. glutamicum* K9 (pEKEx2-YwmC-cutinase), or 1:1 (A), or 1:100 (B) mixtures of both strains. 100,000 cells of each strain or the mixture of strains were analyzed. For all sortings, electronic gating was set in a dot plot of FSC-H against FSC-W to exclude cell doublets and cell debris (gate 0). Selection of cells with respect to the better performing signal peptide was performed by setting a gate in the dot blot (gate 1) that contains as many of the better performing cells (i.e. those with the NprE signal peptide) and that excludes as many of the less productive cells (i.e. those with the YwmC signal peptide) as possible. The number of cells falling into the respective gates are shown

For the FACS sorting, we then selected a gate such that it includes as many of the better performing cells (i.e. 309 cells containing pEKEx2-NprE-cutinase; Table 2A and Additional file 1: Figure S4A) and excludes as many of the less productive cells (i.e. 7 cells containing pEKEx2-YwmC-cutinase; Table 2A and Additional file 1: Figure S4A) as possible (Fig. 5c, gate 1). Next, the two strains were mixed in a ratio of 1:1 and the mixture was analyzed by FACS. In this case, 87 cells now fell into the previously defined sorting gate 1 (Table 2A and Additional file 1: Figure S4A). Hereafter, we performed a single round of enrichment by collecting 5 × 10^4^ cells from gate 1 that were subsequently cultivated in a BioLector for 24 h and then again subjected to FACS analysis. After the enrichment step, FACS analysis indicated that gate 1 now contained 148 cells (Table 2A and Additional file 1: Figure S4A), 46 clones of which were sorted out onto a BHI agar plate containing kanamycin for plasmid identification. DNA sequencing of 44 obtained plasmids revealed that 43 clones (i.e. 97.7%) in fact contained the pEKEx2-NprE-cutinase plasmid (Table 2A). Thus, starting from a 1:1 mixture, a single round of enrichment was sufficient to obtain nearly exclusively cells harboring the plasmid encoding the cutinase precursor protein with the superior NprE signal peptide.

Finally, we asked whether cells secreting the cutinase via the superior NprE signal peptide can also be enriched when the mixture of cells is significantly in favour of those cells which secrete the cutinase via the less effective YwmC signal peptide. Also in this case, the two *C. glutamicum* K9 strains containing pEKEx2-NprE-cutinase or pEKEx2-YwmC-cutinase, respectively, were first cultivated separately and analyzed by FACS (Table 2B). Again, a sorting gate 1 was selected such that it includes as many of the better performing cells (i.e. 458 cells containing pEKEx2-NprE-cutinase) and excludes as many of the less productive cells (i.e. 46 cells containing pEKEx2-YwmC-cutinase) as possible (Table 2B and Additional file 1: Figure S4B). The two strains were then mixed in a ratio of 1:100 (NprE:YwmC). Subsequently, the mixture was again analyzed by FACS and, from this mixture, 50 cells now fell into the previously selected gate 1 (Table 2B and Additional file 1: Figure S4B). A single round of enrichment was then performed by collecting 2 × 10^4^ cells from gate 1. The pooled cells were then cultivated in a BioLector for 24 h and subsequently analyzed by FACS (Table 2B and Additional file 1: Figure S4B). After the enrichment step, now 286 cells analyzed fell into gate 1. Finally, 46 clones were sorted out of the selected gate onto a BHI agar plate containing kanamycin for further analysis. The identity of the signal peptide fused to cutinase in the respective clones was identified by DNA sequencing of the corresponding plasmids. DNA sequencing of 43 obtained plasmids revealed that 16 clones (i.e. 37%) contained the pEKEx2-NprE-cutinase plasmid and 27 clones contained the pEKEx2-YwmC-cutinase plasmid, respectively. These results show that the cells expressing the cutinase with the better performing NprE signal peptide could be significantly enriched from a ratio of 1:100 to a ratio of 1:1.7 (Table 2B), indicating that the K9 secretion biosensor can be used for a FACS-based signal peptide screening, independent of an assay for the desired target protein.

## Discussion

In this study, we have constructed a *C. glutamicum* biosensor strain that allows for the monitoring of Sec-dependent secretion of heterologous proteins in a dose-dependent manner, independent of a direct assay for the desired target protein. By analyzing the cellular transcriptional responses of *C. glutamicum* to the expression and secretion of two heterologous model proteins, the *htrA* gene was found to be upregulated upon high-level secretion of both model proteins. Based on these findings, the *eyfp* gene was integrated into the respective *htrA*-containing five gene operon on the chromosome such that it allows the regulation of *eyfp* expression in a secretion stress-responsive manner. The newly constructed secretion biosensor was able to differentiate between differently efficient signal peptides with respect to the secretion of a cutinase. In addition, we have shown that an efficient signal peptide can be separated from a poor signal peptide by using the biosensor signal of the respective cells in FACS sorting experiments.

Our finding that *htrA* gene expression was upregulated in response to the secretion of different heterologous proteins indicates that, also in *C. glutamicum*, the extracytosolic protease HtrA seems to be a major factor that combats secretion stress by degrading unfolded proteins that accumulate at the membrane-cell envelope interface. Since the amount of misfolded proteins at the *trans*-side of the membrane increases upon increased secretion of proteins via the Sec pathway, the *htrA* gene is upregulated in response to this secretion stress in a dose-dependent manner. It is however completely unknown how this regulation occurs in *C. glutamicum*. For *B. subtilis,* it has been shown that the secretion stress-responsive upregulation of *htrA* (and *htrB*) is mediated by the CssRS two component system [22]. In *M. tuberculosis*, the regulation is more complex and it has been shown that both the extracytoplasmic function (ECF) sigma factor SigE and the two-component system MprAB are involved in the regulation of *htrA* (*pepD*) gene expression in response to various cell envelope stresses [23]. In *M. tuberculosis*, the *htrA* (*pepD*) gene is located in an operon that consists of the genes *mprA*-*mprB*-*pepD*-*moaB2*, a gene organization which is highly similar to that found in *C. glutamicum*, where the *htrA* gene is likewise located in an operon and sandwiched between the genes encoding a two-component system and a *moa* gene (see Fig. 1a). Although not experimentally addressed so far, it therefore seems a likely possibility that the two component system CgtSR2 might be at least one of the factors that are involved in the regulation of *htrA* gene expression in *C. glutamicum*.

In the biosensor strain *C. glutamicum* K9, the *htrA* gene has been replaced by *eyfp* gene and, therefore, the HtrA quality control protease is missing. At a first glance, this might be considered as a disadvantage with respect to the secretory production of heterologous proteins, since accumulated misfolded secreted protein at the *trans*-side of the cytoplasmic membrane can no longer be removed by HtrA. In line with this view, the deletion of *htrA* can have severe negative effects on growth in various microorganisms, especially under protein folding stress conditions such as high temperature [31] and it has also been reported to have negative effects on the secretion of heterologous proteins [24]. However, a direct comparison between *C. glutamicum* wild-type and *C. glutamicum* K9 clearly showed that the K9 strains producing NprE-cutinase (Additional file 1: Figure S5A) or Pre^Lip^-PhoA (Additional file 1: Figure S5B) exhibited even a slightly better growth than the corresponding wild-type strains under standard growth conditions (Additional file 1: Figure S5A and B). This indicates that the secretion stress exerted by these two respective heterologous proteins seems to be rather low under these conditions and that the amounts of accumulated misfolded proteins at the *trans*-side of the membrane do not significantly affect the viability of the cells in a negative way. Furthermore, at least for these two heterologous proteins tested, the yields of active protein obtained with the *C. glutamicum* K9 biosensor strain were equal or even slightly higher than the yields obtained with the *C. glutamicum* wild-type strain (Additional file 1: Figure S5C–E). Similar findings have been reported for *Lactococcus lactis,* which demonstrated that the inactivation of the *htrA* gene can have a beneficial effect on the secretory production of heterologous proteins [32–34]. Nevertheless, the situation may turn in the adverse direction when the secretory production of other heterologous proteins is attempted, especially if those proteins show a poor ability to fold after their membrane translocation, leading to larger amounts of accumulated un/misfolded protein at the membrane-cell wall interface.

The K9 biosensor signal clearly responds to increasing amounts of proteins that are secreted by the Sec pathway across the cytoplasmic membrane in a dose-dependent manner. When NprE-cutinase expression is gradually increased by the addition of increasing amounts of IPTG, the corresponding *C. glutamicum* K9 biosensor cells showed a concomitant increase of both the cutinase activity in the culture supernatant and the specific fluorescence, which holds true also when the IPTG concentration is increased from 50 to 250 µM (Fig. 2c). A slightly different behaviour is observed for *C. glutamicum* K9 expressing Pre^Lip^-PhoA. Here, the upper limit for the formation of active PhoA seems to be reached already at the expression level that is obtained by the addition of 20 µM IPTG, since further increases in IPTG concentration resulted only in minor increases in PhoA activity, although the specific fluorescence of the corresponding cells continuously increased, even at the final step from 50 to 250 µM IPTG (Fig. 3c). In that respect, it is important to note that the secretion biosensor responds to the extent of Sec-dependent protein secretion, but not to correctly folded and therefore biologically active forms of the secreted target proteins. Therefore, the results obtained with Pre^Lip^-PhoA suggest that in the presence of more than 20 µM IPTG, more protein molecules are synthesized and secreted across the plasma membrane (Fig. 3d), but that the cellular capacity to correctly fold and release active PhoA has already reached an upper limit. Since PhoA possesses two disulfide bonds that are required for its activity and stability [35] and which must be formed correctly after its membrane passage, one might speculate that a possible bottleneck for the formation of active PhoA could be that the thiol-disulfide oxidoreductases (TDORs) that are responsible for disulfide bond formation and isomerization in *C. glutamicum* [36, 37] are saturated already at the PhoA expression level that is obtained by 20 µM IPTG. If so, then a further increase of PhoA expression and membrane translocation would result in the accumulation and possibly also release of increasing amounts of misfolded and thereby inactive PhoA protein. Interestingly, the cutinase likewise possesses two disulfide bonds that must be formed after its membrane passage [38]. Nevertheless, as mentioned above, the cutinase activity in the culture supernatant steadily increases with higher IPTG concentrations, even at the final step from 50 to 250 µM IPTG. This might indicate that, in contrast to PhoA secretion, folding after export might be a less severe bottleneck for cutinase secretion. However, an interesting observation in this respect is the finding that the enzymatic activities in the culture supernatants of the NprE-cutinase and YwmC-cutinase expressing strains differ by a factor of two (Fig. 4c), whereas the difference in the amount of secreted cutinase protein seems to be significantly higher (approximately tenfold or even more, Fig. 4d). These findings suggest that a higher secretion level mediated by the more efficient signal peptide (NprE) results in an overproportionate concomitant production of enzymatically inactive protein, indicating that, also for cutinase, an upper limit of the folding capacity seems to exist within the cell. Besides protein folding, also other steps in the secretion pathway, such as the mechanistically to date completely unknown transport of proteins across the mycolic acid outer membrane layer [4], might restrict the amounts of heterologous proteins that can be secreted into the *C. glutamicum* culture supernatant. Furthermore, it is very likely that the bottlenecks in the *C. glutamicum* secretion pathway might differ for different target proteins, depending on their intrinsic folding properties, their requirement for folding factors (such as TDORs), or other, positive (i.e. required for their secretion) or negative interactions between the target proteins and components of the cell envelope.

The identification of an optimal signal peptide for a desired target protein is one of the most critical steps on the way to an efficient secretory production process [12]. Thus far, differentiation between different efficient signal peptides required the availability of an assay for the respective target protein. However, for many biotechnologically or pharmaceutically relevant target proteins, a simple assay that also can be handled for a large number of samples in a high-throughput screening approach is not available. In these cases, the availability of a secretion biosensor that can differentiate between different efficient signal peptides via its fluorescence signal will be of great advantage. As shown in this work, three differently efficient signal peptides for cutinase secretion could clearly be distinguished by the specific fluorescence of the corresponding recombinant *C. glutamicum* K9 biosensor strains.

Furthermore, despite the fact that the difference in the median fluorescence between an efficient (NprE) and a less efficient (YwmC) signal peptide was rather small in the FACS analysis (i.e. 12.1%, see Fig. 5a, b), we could show that, when setting an appropriate sorting gate, the difference in specific fluorescence within the respective single cells is sufficient to allow a significant enrichment of the superior signal peptide out of a 1:1 or even a 1:100 mixture by only one FACS sorting and enrichment step. However, for the separation of signal peptides with even smaller differences in their secretion efficiencies than the two signal peptides examined in this study or for the screening of large signal peptide libraries, two or even more rounds of FACS sorting and enrichment might be required to identify the optimal signal peptide for the given target protein of choice.

## Conclusions

In this study, we have constructed a *C. glutamicum* biosensor strain that allows for the monitoring of Sec-dependent secretion of heterologous proteins in a dose-dependent manner, independent of a direct assay for the respective target protein. The availability of such a biosensor now opens up the way for various applications, such as (1) the screening of large signal peptide libraries for any desired heterologous target protein, (2) the optimization of a given signal peptide by random or saturation mutagenesis approaches, (3) the optimization of the *C. glutamicum* host chassis by directed laboratory evolution, (4) the analysis of population heterogeneities in microfluidic cultivation devices, and (5) the online monitoring of secretory protein production processes.

## Methods

### Bacterial strains, media, and growth conditions

The bacterial strains used in this study are listed in Table 3. *E. coli* DH5α was used as cloning host and grown in LB (lysogeny broth) medium [39] at 37 °C. *C. glutamicum* strains were growth at 30 °C in brain heart infusion medium (BHI, Difco Laboratories), BHIS medium (BHI medium containing 0.5 M sorbitol), CGIII medium [40] or CGXII medium [41] containing 1 to 4% (w/v) glucose as indicated. If required, isopropyl-β-d-thiogalactopyranoside (IPTG) was added to final concentrations between 5 µM and 1 mM as indicated. Antibiotic supplements were at the following concentrations: kanamycin 25 µg ml^−1^ (*C. glutamicum*) and 50 µg ml^−1^ (*E. coli*).

**Table 3:** Bacterial strains and plasmids used in this study

Strains or plasmids	Relevant properties	Source of references
*E. coli* strains		
DH5α	*supE44, ∆lacU169*(ϕ*80 lacZ∆M15*) *hsdR17 recA1 endA1 hsdR gyrA relA thi*	[53]
*C. glutamicum* strains		
ATCC13032	Wild-type	[54]
ATCC13032 K9	htrA::htrA′-eypf (replacement of htrA by htrA′-eyfp)	This study
*C. glutamicum* plasmids		
pSenLys	Lysine sensor plasmid encoding LysG and its target promoter P*lysE* fused to *eyfp*	[44]
pK19mobsacB	Vector for allelic exchange in *C. glutamicum*, pK18 *oriV*_*E.coli*_, *oriT*, *mob*, *sacB*, *lacZ*α, Km^R^	[45]
pK19mobsacB-K9	pK19mobsacB containing the *eyfp* gene flanked by the upstream region and the first 51 bp of the *htrA* structural gene and the *htrA* downstream region	This study
pEKEx2	*C. glutamicum*/*E. coli* shuttle vector for regulated gene expression, pBL1 oriV_*C.glutamicum*_, pUC18 oriV_*E. coli*_, P_*tac*_, *lac*I^q^, Km^R^	[55]
pEKEx2-AmyE	pEKEx2 containing a gene encoding *B. subtilis* AmyE with its authentic signal peptide and a carboxyl-terminal His-tag	This study
pEKEx2-ΔSP-AmyE	pEKEx2 containing a gene encoding a signal peptide-less variant of *B. subtilis* AmyE with a carboxyl-terminal His-tag	This study
pEKEx2-NprE-cutinase	pEKEx2 containing a gene encoding the mature part of the cutinase from *F. solani pisi* fused to the NprE signal peptide	[10]
pEKEx2-YpjP-cutinase	pEKEx2 containing a gene encoding the mature part of the cutinase from *F. solani pisi* fused to the YpjP signal peptide	[10]
pEKEx2-YwmC-cutinase	pEKEx2 containing a gene encoding the mature part of the cutinase from *F. solani pisi* fused to the YwmC signal peptide	[10]

*Km*^*R*^ kanamycin resistance

### Microtiter plate cultivation

Online monitoring of growth and biosensor fluorescence of *C. glutamicum* strains was done in 48-well FlowerPlates (m2p-labs, Aachen/D) using the BioLector micro reactor cultivation device (m2p-labs, Aachen/D). 750 μl CGXII medium containing 2% glucose per well were inoculated with *C. glutamicum* cells from a preculture and grown at 30 °C, a shaking frequency of 1200 rpm, and a shaking diameter of 3 mm. After 4 h of cultivation, the gene expression from pEKEx2-derived plasmids was induced by addition of various concentrations of IPTG as indicated. During the entire cultivation process, the production of biomass was measured as the backscattered light intensity of sent light with a wavelength of 620 nm (signal gain factor of 20) and the eYFP fluorescence of the cultures was measured at an excitation of 508 nm and an emission of 532 nm (signal gain factor of 90). The specific fluorescence for the cells is defined as eYFP fluorescence per scattered light intensity (given in a.u.) [42]. For each experiment, three independent biological replicates were used.

### Plasmid constructions

The plasmids used in this study are listed in Table 3. Oligonucleotides and primers used are listed in Additional file 1: Table S1. All DNA manipulation followed standard procedures [43]. All newly constructed plasmids were verified by DNA sequencing (Eurofins, Ebersberg, Germany). Plasmids pEKEx2-NprE-cutinase, pEKEx2-YpjP-cutinase, and pEKEx2-YwmC-cutinase have been described previously [10]. For the expression of the *B. subtilis* amylase AmyE in *C. glutamicum*, the *amyE* gene was amplified by PCR using chromosomal DNA of *B. subtilis* DB104 as template and primers AmyE-His-fw and AmyE-His-rv. The resulting PCR fragment was purified, digested with *Bam*HI and *Sac*I and ligated into *Bam*H/*Sac*I-digested pEKEx2, resulting in pEKEx2-AmyE. For the expression of a signal peptide less variant of the *B. subtilis* amylase AmyE, the DNA region encoding the mature AmyE protein was amplified by PCR using chromosomal DNA of *B. subtilis* DB104 as template and primers ΔSP-AmyE-His-fw and AmyE-His-rv. The resulting PCR fragment was purified, digested with *Bam*HI and *Sac*I and ligated into *Bam*HI/*Sac*I-digested pEKEx2, resulting in pEKEx2-ΔSP-AmyE.

### Strain constructions

For the construction of *C. glutamicum* K9 containing a replacement of the chromosomal *htrA* gene by a DNA fragment encompassing the first 51 nucleotides of the *htrA* structural gene followed by a TAG stop codon, a 16 bp spacer sequence containing a ribosome binding site, and the *eyfp* gene (Fig. 1b), three different DNA fragments were generated by PCR. The first fragment containing approximately 580 bp of the *htrA* upstream region followed by 51 bp of the *htrA* structural gene and a TAG stop codon (FK9-1) was obtained by using chromosomal DNA of *C. glutamicum* ATCC13032 as template and primers up-fw and up-0998-rv. The second fragment containing the *eyfp* gene preceded by a ribosome binding site (FK9-2) was obtained by using pSenLys [44] as template and primers RBS-eyfp-fw and eyfp-rv. The third DNA fragment (FK9-3) containing approximately 580 bp of the *htrA* downstream region was generated by using chromosomal DNA of *C. glutamicum* ATCC13032 as template and primers dw-fw and dw-rv. The corresponding PCR fragments were purified and fused together in cross-over PCR reactions. First, fragments FK9-1 and FK9-2 were fused using both fragments as template and primers up-fw and eyfp-rv. The resulting fused fragment (FK9-4) was then fused with fragment FK9-3 using FK9-4 and FK9-3 as template and primers up-fw and dw-rv. The resulting DNA fragment FK9-5 was then ligated into *Sma*I-digested pK19mobsacB [45], yielding pK19mobsacB-K9. pK19mobsacB-K9 was introduced into *C. glutamicum* ATCC13032 by electroporation [46] and cells that had integrated the plasmid into the chromosome via homologous recombination were selected on plates containing kanamycin. A second homologous recombination event leading to the loss of the *sacB* gene (via excision of the integrated plasmid) was positively selected on BHIS agar plates containing 10% (w/v) sucrose. Colonies were subsequently analyzed for the successful gene replacement on the chromosome by colony PCR using primers proof-fw and proof-rv. One of the isolates that contained the desired replacement was designated *C. glutamicum* K9.

### Flow cytometry

*Corynebacterium glutamicum* strains were first cultivated in flowerplates in 800 µl CGIII medium containing 2% (w/v) glucose at 900 rpm for 8 h at 30 °C (preculture 1). Subsequently, 100 µl of the respective cultures were added to 700 µl CGXII medium containing 1% (w/v) glucose and grown overnight at 900 rpm and 30 °C (preculture 2). The resulting cultures were then used to inoculate the main cultures (800 µl) in CGXII medium containing 2% (w/v) glucose to an OD_600_ of 0.5. The cultures were then grown in a BioLector at 1200 rpm and 30 °C, and, after 4 h of growth, ITPG was added (250 µM final concentration). After further 6 h of growth, cells from the respective cultures were subjected to FACS analysis or cell sorting.

For the separation of two differently efficient signal peptides out of a 1:1 or a 1:100 mixture of the respective cells by FACS, an enrichment step was performed. For this purpose, 5 × 10^4^ (in the case of the 1:1 mixture) or 2 × 10^4^ cells (in the case of the 1:100 mixture) were sorted out of the preselected gate 1 into 800 µl CGXII medium containing 2% (w/v) glucose and subsequently grown for 24 h in a BioLector at 1200 rpm and 30 °C. The respective culture was then used to inoculate 800 µl CGXII medium containing 2% (w/v) glucose to an OD_600_ of 0.5 and treated further as described above for subsequent FACS analysis. Finally, after the enrichment step, 46 cells were sorted out of the preselected gate onto BHI agar plates containing 25 μg ml^−1^ kanamycin. A graphical workflow of the FACS analysis and sorting experiments is shown in Additional file 1: Figure S3.

Flow cytometric measurements and sorting were performed on a FACS ARIA III high speed cell sorter (BD Biosciences, Franklin Lakes, NJ, USA). A 488 nm blue solid state laser was used for the detection of eYFP fluorescence with a 502-nm long-pass and a 530/30-nm band-pass filter set. Forward scatter (FSC) and side-scatter (SSC) were detected as a voltage pulse of height (H) and width (W). Sorting was performed with a custom four-way purity single-cell precision mode at a threshold rate up to 10^4^ events/s and a sample pressure of 70 psi. For cell sorting, a preselection of cells was performed to exclude cell doublets and debris by electronic gating using FSC-W against FSC-H (gate 0). All FACS data were analyzed using BD DIVA 8.0.1 and FlowJo 10.6.0 software (Tree Star, Inc., Ashland, OR, USA).

### Miscellaneous procedures

Transcriptome analyses by DNA microarray experiments were performed as described by Kleine et al. [20]. Specifically, precultures of the respective *C. glutamicum* strains in CGXII medium containing 4% glucose were grown to an OD_600_ of 5–6 and used to inoculate the main cultures in the same medium to an OD_600_ of 0.5, which subsequently were grown to the mid-exponential growth phase. The main cultures were then splitted into two cultures and IPTG was added to a final concentration of 1 mM to one of the cultures. The cells were harvested 30 min after IPTG addition by pouring the cultures into ice-containing tubes precooled to − 20 °C followed by centrifugation (3 min, 4200×*g*, 4 °C). The cell pellets were quickly frozen in liquid nitrogen and stored at − 80 °C until use for RNA isolation and synthesis of fluorescently labeled cDNA as described by Möker et al. [47]. All DNA microarray analyses were performed with custom-made DNA microarrays based on 70mer oligonucleotides obtained from Operon Biotechnologies. The comparisons were performed in three independent biological replicates. The experimental details for handling these microarrays are described elsewhere [48]. Processed and normalized data as well as experimental details were stored in an inhouse microarray database for further analysis [49]. The full microarray data sets of this study have also been deposited in the NCBI Gene Expression Omnibus and can be found under the GEO Accession number GSE140735. Preparation of whole cell extracts and supernatant fractions of *C. glutamicum* strains for subsequent analysis by SDS-PAGE were performed as described by Meissner et al. [50]. Cutinase activity in the culture supernatant fractions was determined as previously described [51]. Alkaline phosphatase (PhoA) activity in the culture supernatant fractions was determined as previously described by Darmon et al. [52].

## Supplementary information

**Additional file 1: Table S1.** Oligonucleotides and primers used in this study. **Figure S1.** Expression and localization of heterologous proteins in *C. glutamicum* K9. **Figure S2.** *C. glutamicum* wild-type does not possess any significant intrinsic fluorescence. **Figure S3.** Graphic workflow for the FACS analysis and sorting experiments. **Figure S4.** FACS analysis of *C. glutamicum* K9 strains. **Figure S5.** Secretory production of cutinase and PhoA by *C. glutamicum* wild-type and *C. glutamicum* K9 strains.

## Data Availability

All data generated or analysed during this study are included in this published article and its additional files

## References

[1] Quax WJ (1997). Merits of secretion of heterologous proteins from industrial microorganisms. Folia Microbiol.

[2] Lee JY, Na YA, Kim ES, Lee HS, Kim P (2016). The actinobacterium *Corynebacterium glutamicum*, an industrial workhorse. J Microbiol Biotechnol.

[3] Becker J, Gießelmann G, Hoffmann SL, Wittmann C (2018). *Corynebacterium glutamicum* for sustainable bioproduction: from metabolic physiology to systems metabolic engineering. Adv Biochem Eng Biotechnol.

[4] Freudl R (2017). Beyond amino acids: use of the *Corynebacterium glutamicum* cell factory for the secretion of heterologous proteins. J Biotechnol.

[5] Vertès AA, Yukawa H, Inui M (2013). Protein secretion systems of *Corynebacterium glutamicum*. *Corynebacterium glutamicum*. Biology and biotechnology.

[6] Freudl R (2013). Leaving home ain’t easy: protein export systems in Gram-positive bacteria. Res Microbiol.

[7] Rusch SL, Kendall DA (2007). Interactions that drive Sec-dependent bacterial protein transport. Biochemistry.

[8] Denks K, Vogt A, Sacchelaru I, Petriman NA, Kudva R, Koch HG (2014). The Sec translocon mediated protein transport in prokaryotes and eukaryotes. Mol Membr Biol.

[9] Dalbey RE, Wang P, van Dijl JM (2012). Membrane proteases in the bacterial protein secretion and quality control pathway. Microbiol Mol Biol Rev.

[10] Hemmerich J, Moch M, Jurischka S, Wiechert W, Freudl R, Oldiges M (2019). Combinatorial impact of Sec signal peptides from *Bacillus subtilis* and bioprocess conditions on heterologous cutinase secretion by *Corynebacterium glutamicum*. Biotechnol Bioeng.

[11] Brockmeier U, Caspers M, Freudl R, Jockwer A, Noll T, Eggert T (2006). Systematic screening of all signal peptides from *Bacillus subtilis*: a powerful strategy in optimizing heterologous protein secretion in Gram-positive bacteria. J Mol Biol.

[12] Freudl R (2018). Signal peptides for recombinant protein secretion in bacterial expression systems. Microb Cell Fact.

[13] Rohe P, Venkanna D, Kleine B, Freudl R, Oldiges M (2012). An automated workflow for enhancing microbial bioprocess optimization on a novel microbioreactor platform. Microb Cell Fact.

[14] Freier L, Hemmerich J, Schöler K, Wiechert W, Oldiges M, von Lieres E (2016). Framework for Kriging-based iterative experimental analysis and design: optimization of secretory protein production in *Corynebacterium glutamicum*. Eng Life Sci.

[15] Raivio TL, Silhavy TJ (2001). Periplasmic stress and ECF sigma factors. Annu Rev Microbiol.

[16] Meltzer M, Hasenbein S, Mamant N, Merdanovic M, Poepsel S, Hauske P, Kaiser M, Huber R, Krojer T, Clausen T, Ehrmann M (2009). Structure, function and regulation of the conserved serine proteases DegP and DegS of *Escherichia coli*. Res Microbiol.

[17] Hyyryläinen HL, Bolhuis A, Darmon E, Muukkonen L, Koski P, Vitikainen M, Sarvas M, Pragai Z, Bron S, van Dijl JM, Kontinen VP (2001). A novel two-component regulatory system in *Bacillus subtilis* for the survival of severe secretion stress. Mol Microbiol.

[18] Westers H, Westers L, Darmon E, van Dijl JM, Quax WJ, Zanen G (2006). The CssRS two-component regulatory system controls a general secretion stress response in *Bacillus subtilis*. FEBS J.

[19] Trip H, van der Veek PJ, Renniers TC, Maima R, Sagt CM, Mohrmann L, Kuipers OP (2011). A novel screening system for secretion of heterologous proteins in *Bacillus subtilis*. Microb Biotechnol.

[20] Kleine B, Chattopadhyay A, Polen T, Pinto D, Mascher T, Bott M, Brocker M, Freudl R (2017). The three-component system EsrISR regulates a cell envelope stress response in *Corynebacterium glutamicum*. Mol Microbiol.

[21] Yang M, Galizzi A, Henner D (1983). Nucleotide sequence of the amylase gene from *Bacillus subtilis*. Nucleic Acids Res.

[22] Darmon E, Noone D, Masson A, Bron S, Kuipers OP, Devine KM, van Dijl JM (2002). A novel class of heat and secretion stress-responsive genes is controlled by the autoregulated CssRS two-component system of *Bacillus subtilis*. J Bacteriol.

[23] White MJ, He H, Penoske RM, Twining SS, Zahrt TC (2010). PepD participates in the mycobacterial stress response mediated through MprAB and SigE. J Bacteriol.

[24] Vicente RL, Gullón S, Marín S, Mellado RP (2016). The three *Streptomyces lividans* HtrA-like proteases involved in the secretion stress response act in a cooperative manner. PLoS ONE.

[25] Pfeifer-Sancar K, Mentz A, Rückert C, Kalinowski J (2013). Comprehensive analysis of the *Corynebacterium glutamicum* transcriptome using an improved RNAseq technique. BMC Genomics.

[26] Samorski M, Müller-Newen G, Büchs J (2005). Quasi-continuous combined scattered light and fluorescence measurements: a novel measurement technique for shaken microtiter plates. Biotechnol Bioeng.

[27] Glick B (1995). Metabolic load and heterologous gene expression. Biotechnol Adv.

[28] Hamed MB, Anné J, Karamanou S, Economou A (2018). *Streptomyces* protein secretion and its application in biotechnology. FEMS Microbiol Lett.

[29] Götz F, Popp F, Korn E, Schleifer KH (1985). Complete nucleotide sequence of the lipase from *Staphylococcus hyicus* cloned in *Staphylococcus carnosus*. Nucleic Acids Res.

[30] Kouwen TRHM, Nielsen AK, Denham EL, Dubois JYF, Dorenbos R, Rasmussen MD, Quax WJ, Freudl R, van Dijl JM (2010). Contributions of the pre- and pro-regions of a *Staphylococcus hyicus* lipase to secretion of a heterologous protein by *Bacillus subtilis*. Appl Environ Microbiol.

[31] Hansen G, Hilgenfeld R (2013). Architecture and regulation of HtrA-family proteins involved in protein quality control and stress response. Cell Mol Life Sci.

[32] Miyoshi A, Poquet I, Azevedo V, Commissaire J, Bermudez-Humaran L, Domakova E, Le Loir Y, Oliveira SC, Gruss A, Langella P (2002). Controlled production of stable heterologous proteins in *Lactococcus lactis*. Appl Environ Microbiol.

[33] Cortes-Perez NG, Poquet I, Oliveira M, Gratadoux JJ, Madsen SM, Miyoshi A, Corthier G, Azevedo V, Langella P, Bermudez-Humaran LG (2006). Construction and characterization of a *Lactococcus lactis* strain deficient in intracellular ClpP and extracellular HtrA proteases. Microbiology.

[34] Morello E, Bermudez-Hamaran LG, Llull D, Sole V, Miraglio N, Langella P, Poquet I (2008). *Lactococcus lactis*, an efficient cell factory for recombinant protein production and secretion. J Mol Microbiol Biotechnol.

[35] Sone M, Kishigami S, Yoshihisa T, Ito K (1997). Roles of disulfide bonds in bacterial alkaline phosphatase. J Biol Chem.

[36] Dutton RJ, Boyd D, Berkmen M, Beckwith J (2008). Bacterial species exhibit diversity in their mechanisms and capacity for protein disulfide bond formation. Proc Natl Acad Sci USA.

[37] Daniels R, Mellroth P, Bernsel A, Neiers F, Normark S, von Heijne G, Henriques-Normark B (2010). Disulfide bond formation and cysteine exclusion in gram-positive bacteria. J Biol Chem.

[38] Longhi S, Cambillau C (1999). Structure-activity of cutinase, a small lipolytic enzyme. Biochim Biophys Acta.

[39] Bertani G (1951). Studies on lysogenesis. I. The mode of phage liberation by lysogenic *Escherichia coli*. J Bacteriol.

[40] Menkel E, Thierbach G, Eggeling L, Sahm H (1989). Influence of increased aspartate availability on lysine formation by a recombinant strain of *Corynebacterium glutamicum* and utilization of fumarate. Appl Environ Microbiol.

[41] Keilhauer C, Eggeling L, Sahm H (1993). Isoleucine synthesis in *Corynebacterium glutamicum*: molecular analysis of the *ilvB*-*livN*-*ilvC* operon. J Bacteriol.

[42] Kensy F, Zang E, Faulhammer C, Tan RK, Büchs J (2009). Validation of a high-throughput fermentation system based on online monitoring of biomass and fluorescence in continuously shaken microtiter plates. Microb Cell Fact.

[43] Sambrook J, MacCallum P, Russel D (2001). Molecular cloning. A laboratory manual.

[44] Binder S, Schendzielorz G, Stäbler N, Krumbach K, Hoffmann K, Bott M, Eggeling L (2012). A high-throughput approach to identify genomic variants of bacterial metabolite producers at the single-cell level. Genome Biol.

[45] Schäfer A, Tauch A, Jäger W, Kalinowski J, Thierbach G, Pühler A (1994). Small mobilizable multi-purpose cloning vectors derived from the *Escherichia coli* plasmids pK18 and pK19: selection of defined deletions in the chromosome of *Corynebacterium glutamicum*. Gene.

[46] van der Rest ME, Lange C, Molenaar D (1999). A heat shock following electroporation induces highly efficient transformation of *Corynebacterium glutamicum* with xenogeneic plasmid DNA. Appl Microbiol Biotechnol.

[47] Möker N, Brocker M, Schaffer S, Krämer R, Morbach S, Bott M (2004). Deletion of the genes encoding the MtrA–MtrB two-component system of *Corynebacterium glutamicum* has a strong influence on cell morphology, antibiotics susceptibility and expression of genes involved in osmoprotection. Mol Microbiol.

[48] Brocker M, Schaffer S, Mack C, Bott M (2009). Citrate utilization by *Corynebacterium glutamicum* is controlled by the CitAB two-component system through positive regulation of the citrate transport genes *citH* and *tctCBA*. J Bacteriol.

[49] Polen T, Wendisch VF (2004). Genomewide expression analysis in amino acid-producing bacteria using DNA microarrays. Appl Biochem Biotechnol.

[50] Meissner D, Vollstedt A, van Dijl JM, Freudl R (2007). Comparative analysis of twin-arginine (Tat)-dependent protein secretion of a heterologous model protein (GFP) in three different Gram-positive bacteria. Appl Microbiol Biotechnol.

[51] Caspers M, Brockmeier U, Degering C, Eggert T, Freudl R (2010). Improvement of Sec-dependent secretion of a heterologous model protein in *Bacillus subtilis* by saturation mutagenesis of the N-domain of the AmyE signal peptide. Appl Microbiol Biotechnol.

[52] Darmon E, Dorenbos R, Meens J, Freudl R, Antelmann H, Hecker M, Kuipers OP, Bron S, Quax WJ, Dubois JYF, van Dijl JM (2006). A disulfide bond-containing alkaline phosphatase triggers a BdbC-dependent secretion stress response in *Bacillus subtilis*. Appl Environ Microbiol.

[53] Hanahan D (1983). Studies on transformation of *Escherichia coli* with plasmids. J Mol Biol.

[54] Kinoshita S, Udaka S, Shimono M (1957). Studies on amino acid fermentation. Part I. Production of l-glutamic acid by various microorganisms. J Gen Appl Microbiol.

[55] Eikmanns BJ, Thumschmitz N, Eggeling L, Lüdtke KU, Sahm H (1994). Nucleotide sequence, expression and transcriptional analysis of the *Corynebacterium glutamicum gltA* gene encoding citrate synthase. Microbiology.

