# Supplementary material for: A secretion biosensor for monitoring Sec-dependent protein export in Corynebacterium glutamicum
Source: Microb Cell Fact. 2020 Jan 21;19:11. doi: 10.1186/s12934-019-1273-z (PMC6975037; doi:10.1186/s12934-019-1273-z)
Supplement: Supplementary file 1 — Additional file 1: Table S1. Oligonucleotides and primers used in this study. Figure S1. Expression and localization of heterologous proteins in C. glutamicum K9. Figure S2. C. glutamicum wild-type does not possess any significant intrinsic fluorescence. Figure S3. Graphic workflow for the FACS analysis and sorting experiments. Figure S4. FACS analysis of C. glutamicum K9 strains. Figure S5. Secretory production of cutinase and PhoA by C. glutamicum wild-type and C. glutamicum K9 strains. [file 12934_2019_1273_MOESM1_ESM.pdf]

Additional file

**A secretion biosensor for monitoring Sec-dependent protein export in *Corynebacterium glutamicum***

by S. Jurischka, A. Bida, D. Dohmen-Olma, B. Kleine, J. Potzkei, S. Binder, G. Schaumann, P. J. Bakkes, and R. Freudl

<u>Contents:</u>

Table S1. Oligonucleotides and primers used in this study.

Figure S1. Expression and localization of heterologous proteins.

Figure S2. *C. glutamicum* wild-type does not possess a significant intrinsic fluorescence.

Figure S3. Graphic workflow for the FACS analysis and sorting experiments.

Figure S4. FACS analysis of *C. glutamicum* K9 strains.

Figure S5. Secretory production of cutinase and PhoA by *C. glutamicum* wild-type and *C. glutamicum* K9 strains.

## Table S1. Oligonucleotides and primers used in this study

| Name | Sequence (5’ → 3’) |
|---|---|
| **up-fw** | GAAGAAACCGCCGAAACGTCAAGC |
| **eyfp-rv** | GCAGATTAACTCTTTTAAACTTATTACTTGTACAGCTCGTCCATGCCG |
| **dw-fw** | GTTTAAAAGAGTTAATCTGCATCTAATCAAGTAGCC |
| **dw-rv** | GCCATCACGAATTGCCGAACGAG |
| **up-0998-rv** | CGATGCACGGTCCGGGTTCTC |
| **RBS-eyfp-fw** | GAGAACCCGGACCGTGCATCGTAGAAGAAGGAGATATCATATGG |
| **proof-fw** | GTTGATGGCAACCAACAGTG |
| **proof-rv** | CTCGTCTACGTTGTCTACTC |
| **AmyE-His-fw** | GAGGGGATCCCGAAGGAGATATAGATATGTTTGCAAAACGATTCA |
| **ΔSP-AmyE-His-fw** | CTAGAGGATCCCGAAGGAGATATAGATATGGAAACGGCGAACAAATC |
| **AmyE-His-rv** | GATCTAGAGCTCCTAGTGATGGTGATGGTGATGATGGGGAAGAGAACCGCTTAA |

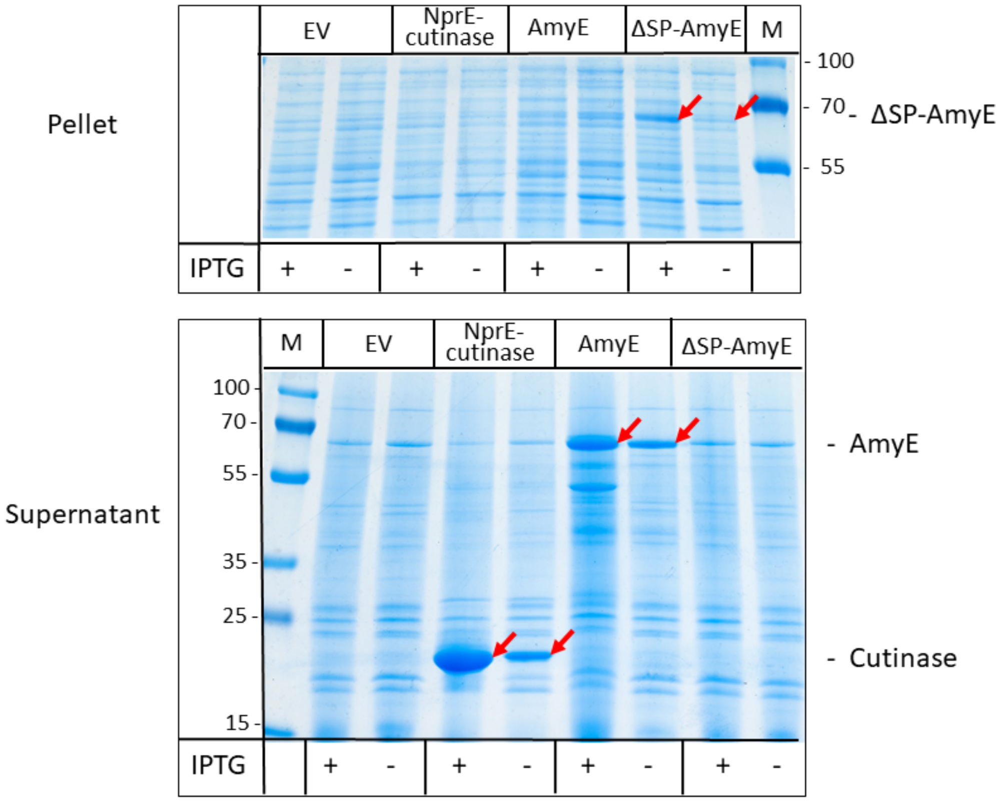


**Figure S1.** Expression and localization of heterologous proteins in *C. glutamicum* K9. Cultures of *C. glutamicum* K9 strains harboring the pEKEx2 empty vector (EV), pEKEx2-AmyE (AmyE), pEKEx2-ΔSP-Amy (ΔSP-Amy), or pEKEx2-NprE-cutinase (NprE-Cutinase) that were grown in the presence (+) or absence (-) of 250 µM IPTG were fractionated into cells (pellet) and culture supernatant. Samples of both fractions corresponding to an equal number of cells were analyzed by SDS-PAGE and proteins were visualized by Commassie Brillant Blue staining. The positions of the different heterologous proteins within the respective fractions are marked by red arrows and are indicated on the right margin. M, molecular weight protein markers in kDa.

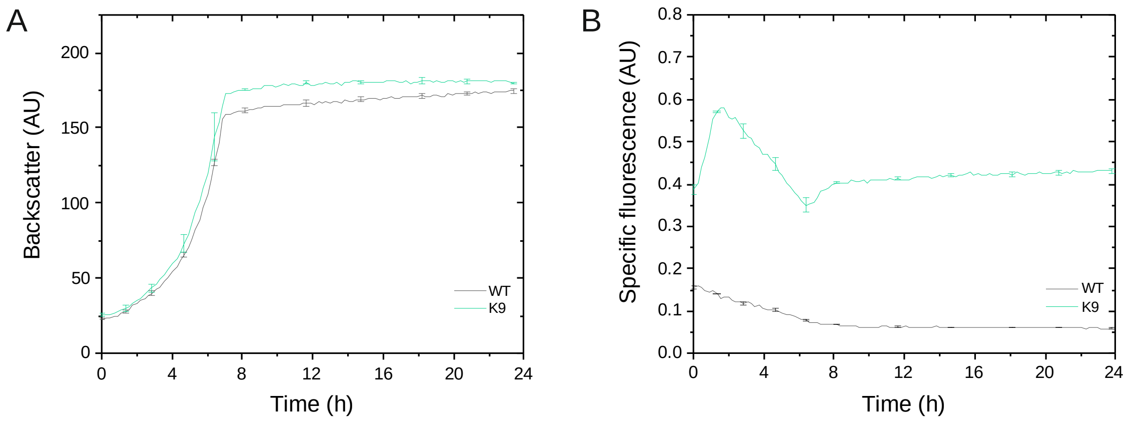


**Figure S2.** *C. glutamicum* wild-type does not possess a significant intrinsic fluorescence. Cells of *C. glutamicum* wild-type (WT) or *C. glutamicum* K9 (K9) were inoculated to an $OD_{600}$ of 0.5 in 750 µl CGXII medium in a 48-well FlowerPlate and subsequently cultivated in a BioLector system for 24 h at 30°C, 1200 rpm and constant 85% relative humidity. (A) Growth of the respective cultures was monitored as backscattered light in 15 min intervals starting at the beginning of the cultivation. The growth curves show one representative experiment of three independent biological replicates. Standard deviations are given for selected time points. (B) Specific fluorescence of the respective cultures during the BioLector cultivation. Also here, one representative experiment of three independent biological replicates is shown and the standard deviations are given for selected time points.

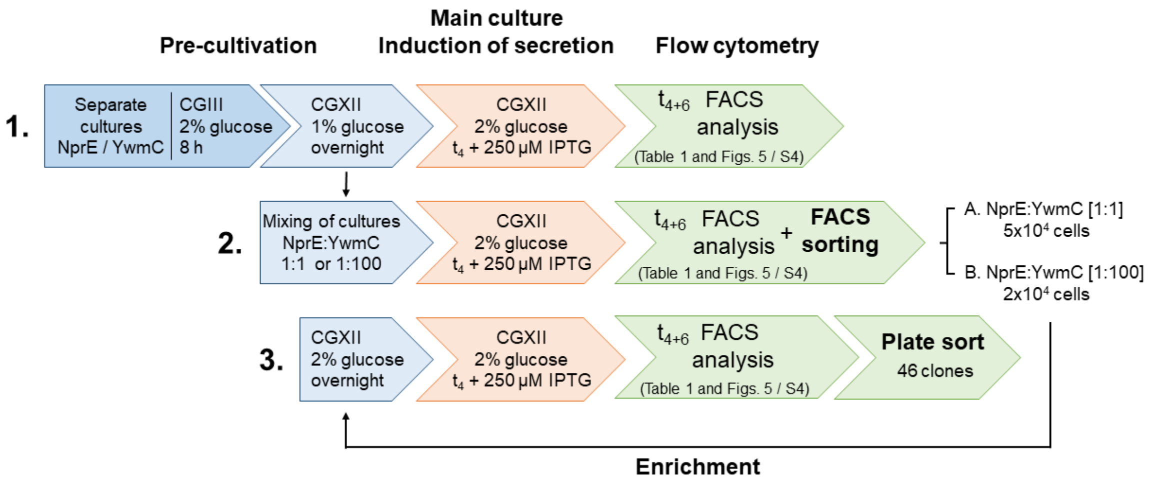


**Figure S3.** Graphical workflow for the FACS analysis and sorting experiments.

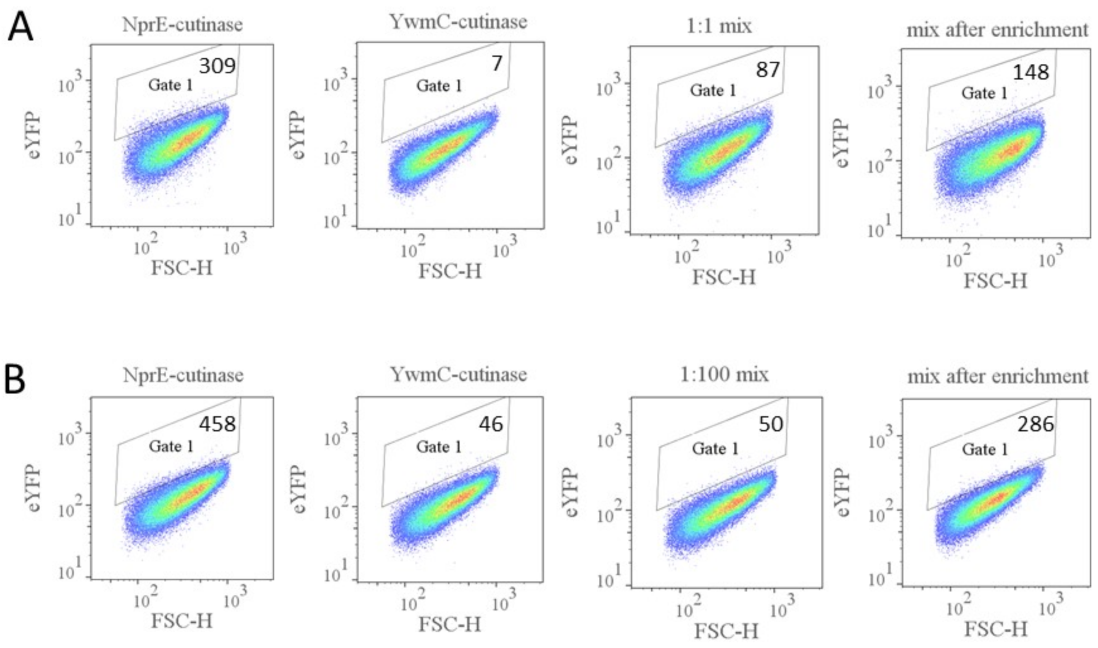


**Figure S4.** FACS analysis of *C. glutamicum* K9 strains. The strains used in the experiments were *C. glutamicum* K9 (pEKEx2-NprE-cutinase), *C. glutamicum* K9 (pEKEx2-YwmC-cutinase), or 1:1 (A) or 1:100 (B) mixtures of both strains. 100 000 cells of each strain or the mixture of strains were analyzed. To exclude doublets and debris, cells were preselected by electronic gating using FSC-W against FSC-H. Selection of cells with respect to the better performing signal peptide was performed by setting a gate in the dot blot (gate 1) that contains as many of the better performing cells (*i.e.* those with the NprE signal peptide) and that excludes as many of the less productive cells (*i.e.* those with the YwmC signal peptide) as possible. The number of cells falling into the respective gates 1 are indicated in the upper right corner (see also Table 2 in the main manuscript).

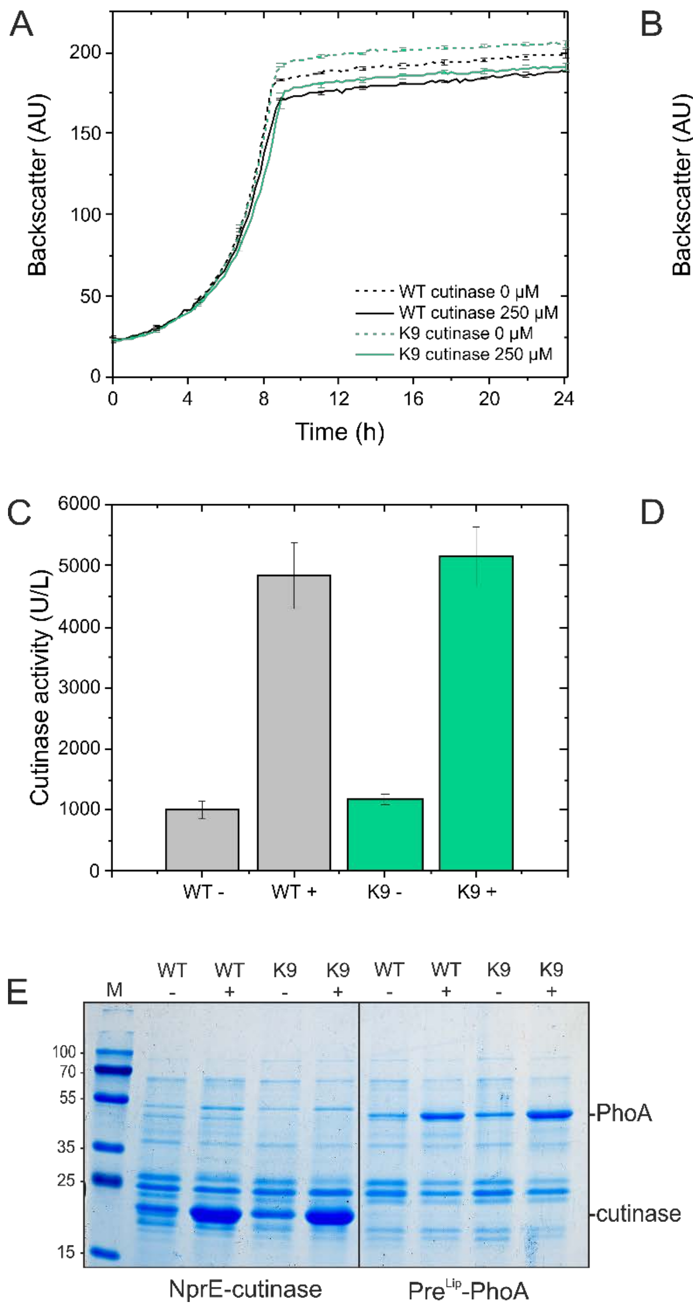


**Figure S5.** Secretory production of cutinase and PhoA by *C. glutamicum* wild-type and *C. glutamicum* K9 strains. Cells of *C. glutamicum* wild-type (WT) or *C. glutamicum* K9 containing (A) pEKEx2-NprE-cutinase (cutinase) or (B) pEKEx2-Pre$^{Lip}$-PhoA (PhoA) were inoculated to an $OD_{600}$ of 0.5 in 750 µl CGXII medium in a 48-well FlowerPlate and subsequently cultivated in a BioLector system for 24 h at 30°C, 1200 rpm and constant 85% relative humidity. For the full induction of gene expression, IPTG was added to a 250 µM final concentration (+). Uninduced cells showing a basal level of gene expression received no IPTG (-). Growth of the respective cultures in (A) and (B) was monitored as backscattered

light in 15 min intervals. The growth curves show one representative experiment of three independent biological replicates. Standard deviations are given for selected time points. (C) Cutinase activity in the supernatant of *C. glutamicum* WT and K9 cells harboring pEKEx2-NrpE-cutinase after 24 h of cultivation with (+) or without (-) added IPTG. (D) PhoA activity in the supernantant of *C. glutamicum* WT and K9 cells harboring pEKEx2-Pre$^{Lip}$-PhoA after 24 h of cultivation with (+) or without (-) added IPTG. (E) Supernatant fractions of *C. glutamicum* WT or K9 cells harboring pEKEx2-NprE-cutinase or pEKEx2-Pre$^{Lip}$-PhoA after 24 h of cultivation with (+) or without (-) added IPTG were analyzed by SDS-PAGE and proteins were visualized by Coomassie Brillant Blue straining. The positions of the secreted cutinase and PhoA proteins are indicated. M, molecular weight protein markers in kDa.
